# Editorial: Exploring the Crosstalk Between Adipose Tissue and the Cardiovascular System

**DOI:** 10.3389/fcell.2022.973135

**Published:** 2022-07-15

**Authors:** Ileana Badi, Elena Sommariva, Kazuo Miyazawa, Hidekazu Kondo, Valerio Azzimato, Nadia Akawi

**Affiliations:** ^1^ Division of Cardiovascular Medicine, University of Oxford, Oxford, United Kingdom; ^2^ Unit of Vascular Biology and Regenerative Medicine, Centro Cardiologico Monzino IRCCS, Milan, Italy; ^3^ Laboratory for Cardiovascular Genomics and Informatics, RIKEN Center for Integrative Medical Sciences, Yokohama, Japan; ^4^ Department of Cardiology and Clinical Examination, Faculty of Medicine, Oita University, Yufu, Japan; ^5^ Center for Infectious Medicine, Department of Medicine Huddinge, Karolinska Institute, Karolinska University Hospital, Stockholm, Sweden; ^6^ Department of Genetics and Genomics, College of Medicine and Health Sciences, United Arab Emirates University, Al-Ain, United Arab Emirates

**Keywords:** adipose tissue, cardiovascular system, cardiovascular disease, adipokines, obesity

Many studies have confirmed the existence of a bidirectional cross-talk between adipose tissue (AT) and the cardiovascular system, which is crucial to the maintenance of normal physiological function in both tissues (Oikonomou and Antoniades, 2019). AT can be considered an endocrine organ *per se* releasing signaling messengers, such as microvesicles and immunomodulatory factors, that influence the development and progression of cardiovascular diseases (CVD) (Oikonomou and Antoniades, 2019). For instance, the altered structure and function of AT in obesity is accompanied by a dysregulated profile of adipokine secretion and is closely associated with an increased incidence of adverse CVD outcomes (Oikonomou and Antoniades, 2019). Similarly, the cardiovascular system secretes molecules that are detected by the surrounding AT, which “senses” cardiovascular inflammation and modify its secretory profile accordingly (Oikonomou and Antoniades, 2019).

The objective of this Research Topic was to receive studies that describe AT dysfunction in the metabolic syndrome and to delineate the role of AT-derived immunomodulatory factors in the development and progression of CVD. We also shed light on the role of “inside-to-outside” signaling (from the cardiovascular system to AT) in the cross-talk between the myocardium, the vasculature, and AT. Eight submissions were received (two reviews, one perspective and five research articles).

In this Research Topic, two review articles highlighted the role of different molecules in this crosstalk. Shalaby et al. reviewed the detrimental role of ceramides in the pathogenesis of cardiometabolic disorders including CVD, type II diabetes and obesity. In this paper the authors discussed the mechanisms by which ceramides affect endothelial dysfunction (Akawi et al., 2021). Ceramides were also reported to exert their detrimental effects via increasing insulin resistance, dysregulating cardiac muscle dilation and contractility, and augmenting inflammation. They also provided an update regarding possible ceramide-targeted therapies. Soci et al. instead described the current knowledge on the epigenetic role exerted by microRNAs in the cross-talk between AT and the cardiovascular system. They also discussed the latest evidence for heart-enriched microRNAs in cardiovascular diseases as epigenetic biomarkers, which regulate cardiac- and metabolic-related gene expression. This suggests a clinical utility for microRNAs given their predictive and therapeutic potential for cardiovascular diseases.

Four articles examined the mechanisms through which dysregulated AT can affect CVD development. Piacentini et al. focused on how innate and adaptive immune cells, present in the perivascular AT (PVAT) of abdominal aortic aneurisms (AAAs), help to sustain a deleterious inflammatory loop. They speculated that trained immunity, which is defined as a form of innate immune memory resulting in enhanced responsiveness to repeated triggers, can be induced and shaped in myeloid progenitor cells by specific innate stimuli and epigenetic and metabolic reprogramming events. This concept was supported by a bioinformatic analysis performed by the authors on AAA human samples and by data from the literature. PVAT also plays an important role in atherosclerosis. For instance, in obesity it promotes the initial step of endothelial dysfunction through mechanisms involving increased oxidative stress and proinflammatory cytokines production (Ketonen et al., 2010). The consequent generation of excess reactive oxygen species and low-density lipoprotein (LDL) oxidation contribute to the progression of atherosclerosis (Poznyak et al., 2021). Li et al. found a mechanistic association in endothelial cells between levels of oxidized LDL (oxLDL) and Neogenin 1 (Neo-1), an inflammatory regulator (Schlegel et al., 2018). OxLDL induced the transactivation of Neo-1 in endothelial cells via enhancing the interaction between CREB1 and BAF47 transcription factors. The authors also revealed that Neo-1 promotes leukocyte adhesion to endothelial cells by modulating the proinflammatory transcription factor NF-κB. Antibody-mediated inhibition of endogenous Neo-1 in mice significantly and markedly reduced atherosclerotic lesions. Using a Mendelian randomization (MR) analysis approach, Huang et al. revealed significant genetic associations between the predicted mass of visceral adiposity and a wide range of traditional metabolic risk factors and endpoints for CVD, including coronary heart disease, cardiac arrhythmias, vascular diseases, and stroke. The authors also conducted a network MR analysis to identify the potential mechanisms linking visceral AT (VAT) with the pathogenesis of CVD. Proteins mainly involved in energy metabolism, inflammation and angiogenesis were highlighted as potential players in the cross-talk between VAT and CVD. Cabaro et al. investigated the paracrine effect of epicardial AT (EAT)-secreted IL-1β on post-operative atrial fibrillation (POAF). They found that patients with POAF had higher IL-1β levels in EAT conditioned medium when compared to the no-POAF group. Furthermore, human atrial fibroblasts exposed to IL-1β and IL-18 showed increased proliferation and migration as well as increased expression levels of proinflammatory and profibrotic genes.

AT can be classified as white AT (WAT) or brown AT (BAT) depending on its metabolic, morphologic, and broader biological phenotype (Badi and Antoniades, 2021). BAT exerts protective effects on cardiometabolic health as well as on the heart (Badi and Antoniades, 2021). Two research articles in this collection focused on understanding the biology of BAT, and WAT browning. Lin et al. observed in a mouse model that the survival of fat grafts for soft-tissue reconstruction is affected by the spontaneous browning of WAT *in situ*. They showed that the local post-transplantation browning of WAT is accompanied by early angiogenesis and superior final graft retention. They proposed that avascularity and hypoxia during the early stage of fat transplantation promote the browning of white adipocytes leading to early revascularization. They also highlighted the beneficial clinical use of browning agents such as CL316243 (Danysz et al., 2018) in improving fat engraftment. Finally, Shaw et al. demonstrated that the beige adipocyte stimulator irisin promotes the release of a novel adipokine, CXCL1, via upregulation of the NF-κB pathway in adipocytes derived from the neck area (which is one of the main sources of BAT). Interestingly, CXCL1 exerted a positive effect on the adhesion of endothelial cells suggesting a possible role for this adipokine in improving tissue vascularization.

Taken together, the articles published within this Research Topic highlighted the importance of deepening our knowledge of the cross-talk between AT and the cardiovascular system in order to assist the development of new and effective therapies for the leading cause of death, CVD.

## References

[B1] Akawi N. Checa A. Antonopoulos A. S. Akoumianakis I. Daskalaki E. Kotanidis C. P. (2021). Fat-secreted Ceramides Regulate Vascular Redox State and Influence Outcomes in Patients with Cardiovascular Disease. J. Am. Coll. Cardiol. 77, 2494–2513. 10.1016/j.jacc.2021.03.314 34016263 PMC8141611

[B2] Badi I. Antoniades C. (2021). Brown Adipose Tissue and the Take (12,13-di)HOME Message to the Heart. Circulation 143, 160–162. 10.1161/CIRCULATIONAHA.120.051981 33428434

[B3] Danysz W. Han Y. Li F. Nicoll J. Buch P. Hengl T. (2018). Browning of White Adipose Tissue Induced by the SS3 Agonist CL-316,243 after Local and Systemic Treatment - PK-PD Relationship. Biochimica Biophysica Acta (BBA) - Mol. Basis Dis. 1864, 2972–2982. 10.1016/j.bbadis.2018.06.007 29902549

[B4] Ketonen J. Shi J. Martonen E. Mervaala E. (2010). Periadventitial Adipose Tissue Promotes Endothelial Dysfunction via Oxidative Stress in Diet-Induced Obese C57Bl/6 Mice. Circ. J. 74, 1479–1487. 10.1253/circj.cj-09-0661 20526041

[B5] Oikonomou E. K. Antoniades C. (2019). The Role of Adipose Tissue in Cardiovascular Health and Disease. Nat. Rev. Cardiol. 16, 83–99. 10.1038/s41569-018-0097-6 30287946

[B6] Poznyak A. V. Nikiforov N. G. Markin A. M. Kashirskikh D. A. Myasoedova V. A. Gerasimova E. V. (2021). Overview of OxLDL and its Impact on Cardiovascular Health: Focus on Atherosclerosis. Front. Pharmacol. 11, 613780. 10.3389/fphar.2020.613780 33510639 PMC7836017

[B7] Schlegel M. Körner A. Kaussen T. Knausberg U. Gerber C. Hansmann G. (2018). Inhibition of Neogenin Fosters Resolution of Inflammation and Tissue Regeneration. J. Clin. Invest. 128, 4711–4726. 10.1172/JCI96259 30222138 PMC6159953

